# The Influence of Spirodi(Iminohydantoin) on Charge Transfer through ds-DNA Containing 8-OXO-dG: A Theoretical Approach

**DOI:** 10.3390/ijms24108570

**Published:** 2023-05-10

**Authors:** Boleslaw T. Karwowski

**Affiliations:** DNA Damage Laboratory of Food Science Department, Faculty of Pharmacy, Medical University of Lodz, ul. Muszynskiego 1, 90-151 Lodz, Poland; boleslaw.karwowski@umed.lodz.pl

**Keywords:** spirodi(iminohydantoin), 7,8-dihydro-8-oxo-2′-deoxyguanosine, clustered DNA damage, charge transfer, DFT

## Abstract

Genetic information stored in a DNA base sequence is continuously exposed to harmful factors. It has been determined that 9 × 10^4^ different DNA damage events occur in a single human cell every 24 h. Of these, 7,8-dihydro-8-oxo-guanosine (^OXO^G) is one of the most abundant and can undergo further transformations towards spirodi(iminohydantoin) (Sp). Sp is highly mutagenic in comparison to its precursor if not repaired. In this paper, the influence of both Sp diastereomers 4*R* and 4*S* as well as their *anti* and *syn* conformers on charge transfer through the double helix was taken into theoretical consideration. In addition, the electronic properties of four modelled double-stranded oligonucleotides (ds-oligos) were also discussed, i.e., d[A_1_Sp_2_A_3_^oxo^G_4_A_5_] * [T_5_C_4_T_3_C_2_T_1_]. Throughout the study, the M06—2X/6—31++G** level theory was used. Solvent–solute non-equilibrated and equilibrated interactions were also considered. The subsequent results elucidated that the 7,8-dihydro-8-oxo-guanosine:cytidine (^OXO^GC) base pair is the settled point of a migrated radical cation in each of the discussed cases, due to its low adiabatic ionization potential, i.e., _~_5.55 [eV]. The opposite was noted for excess electron transfer through ds-oligos containing *anti* (*R*)-Sp or *anti* (*S*)-Sp. The radical anion was found on the ^OXO^GC moiety, whereas in the presence of *syn* (*S*)-Sp or *syn* (*R*)-Sp, an excess electron was found on the distal A_1_T_5_ or A_5_T_1_ base pair, respectively. Furthermore, a spatial geometry analysis of the discussed ds-oligos revealed that the presence of *syn* (*R*)-Sp in the ds-oligo caused only a slight deformation to the double helix, while *syn* (*S*)-Sp formed an almost ideal base pair with a complementary dC. The above results are in strong agreement with the final charge transfer rate constant, as calculated according to Marcus’ theory. In conclusion, DNA damage such as spirodi(iminohydantoin), especially when becoming part of clustered DNA damage, can affect the effectiveness of other lesion recognition and repair processes. This can lead to the acceleration of undesired and deleterious processes such as carcinogenesis or aging. However, in terms of anticancer radio-/chemo- or combined therapy, the slowing down of the repair machinery can result in increased effectiveness. With this in mind, the influence of clustered damage on charge transfer and its subsequent effect on single-damage recognition by glycosylases justifies future investigation.

## 1. Introduction

**Figure 1:**
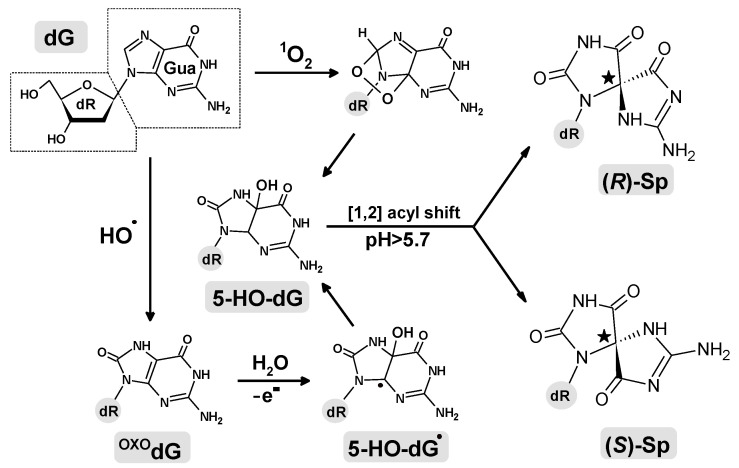
Graphical representation of spirodi(iminohydantoin): two diastereomers (*R*)-Sp and (*S*)-Sp formation in physiological conditions.

The secret of a species’ life is stored in the sequence of nucleic acid base 3 × 10^9^ [1]. Even though the genome is located deep within a cell as highly condensed chromatin and covered by a nucleus layer, it is continuously exposed to harmful factors, both extra- or intra- cellular, such as ionization radiation, xenobiotics, metabolic products, reactive oxygen/nitrogen species, etc. [2,3]. Their activity induces DNA damage, which, if not repaired, can change the genetic information and lead to mutation, carcinogenesis, or cellular aging [4]. It has been established that in each of the 10^14^ human body cells, ~9 × 10^4^ DNA damage events are caused over 24 h [5]. To date, more than 80 types of DNA lesions have been identified [6,7]. According to their mutual proximity, these lesions can be defined as isolated, clustered, or, in special cases, tandem lesions [8]. Because of the huge number of lesions, genetic information is constantly verified and corrected by different repair systems, base/nucleotide excision, homologous recombination and non-homologous end joining [9,10]. Of all the canonical nucleosides, 2’-deoxyguanosine (dG) is most easily one-electron-oxidized thanks to its low ionization potential [11]. The formed dG radical cation can be converted to dG, or after reaction with a water molecule, to 7,8-dihydro-8-oxo-2′-deoxyguanosine (^OXO^dG), one of the most abundant DNA lesions [12]. ^OXO^dG can be further converted to various subsequent products, such as 2′-deoxyspirodi(iminohydantoin) (Sp) (Figure 1) [13]. It should be pointed out here that ^OXO^dG shows a low mutagenicity at a level of 3%, while the mutagenic potential of its derivatives is almost as high as 100% [14]. Sp appearing in the genome can lead to transversion G→T or G→C in the replication step [15]. Barton recently proposed a theory that a glycosylase (e.g., MutY) utilizes an electron transfer to scan the double helix to detect damage in a highly effective way [16]. Therefore, the formation of clustered DNA lesions containing ^OXO^dG and Sp can pose a challenge for the above proteins, which search and excise lesions in the sea of canonical nucleobases [17]. Furthermore, other proteins containing [4Fe-4S] clusters, such as polymerases, helicases, and primase, are communicated by charge transfer through ds-DNA and their activity is suitably regulated and organized. With this in mind, the influence of clustered DNA lesions (CDLs) on electronic properties, structural ds-DNA changes, and charge transfer merits fuller investigation. This should lead to a better understanding of the role of CDLs in the repair and replication of genetic information [9,18]. Additionally, because the effectiveness of most anticancer therapies (radiotherapy, photodynamics, chemotherapy, etc.) is based on DNA damage induction in the targeted cells’ genome, studying the charge transfer process through the double helix should result in the increased safety of therapies by reducing genotoxicity in normal, undamaged cells [19,20,21].

## 2. Results and Discussion

Single- or double-stranded oligonucleotides present in a cell are constantly exposed to the activity of harmful factors, which causes base or sugar moiety injury. Research has found that base modifications induced by the activity of carbonate radicals are over 2000 times more likely to occur than reactions over 2-deoxyribose [22]. 2′-deoxyguanosine has the lowest reduction potential of all the nucleosides (1.29 V/NHE) and thus is the most easily oxidized by ionization radiation, HO^●−^, ^1^O_2_, O_2_^●−^, and other factors [11]. It should be pointed out that in a ds-DNA structure, π-π stacking interaction between neighboring base pairs (BP) allows radical cation migration through the double helix and oxidation at the remote site, usually containing several guanines (GG, GGG, etc.). The above processes can result in 7,8-dihydro-8-oxo-2′-deoxyguanosine formation, which shows a reduction potential lower than dG, i.e., 0.74V/NHE [23]. The ^OXO^dG can be further converted to different oxidation products depending on pH and temperature [24,25]. 

**Figure 2:**
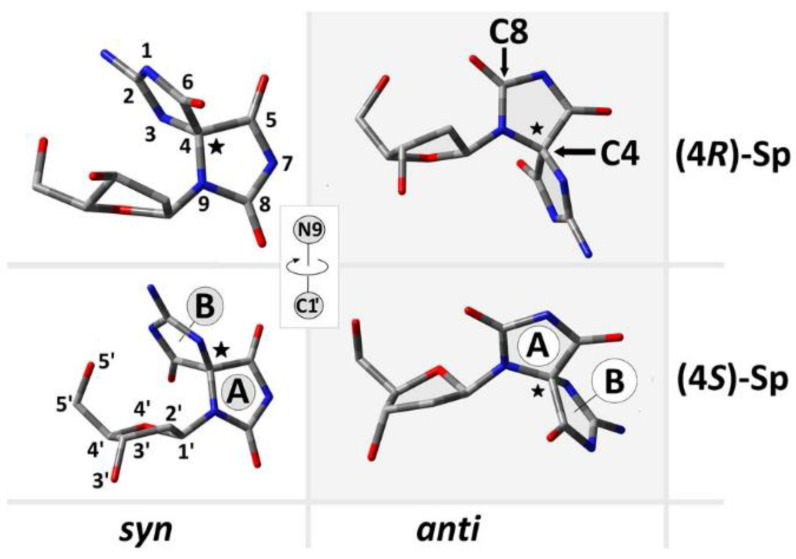
Graphical representation of spirodi(iminohydantoin) diastereomeric forms, isolated from the suitable ds-oligo structures presented in Table 1.

As shown in Figure 1, ^OXO^dG can be further oxidized to spirodi(iminohydantoin). The second important path of Sp formation is the direct dG reaction with singlet oxygen (^1^O_2_). ^1^O_2_ can be the product of photosensitizer Type II activity or can be generated enzymatically by leukocytes [26,27]. Both mechanisms of Sp formation go through the same intermediate, 5-OH-^OXO^dG (Figure 1). The mechanism of Sp formation has been investigated by Burrows, Schlegel, and others. [26,28,29]. Irrespective of the nucleoside substrate type, nucleotide, and single- or double-stranded DNA, spirodi(iminohydantoin) is formed as a mixture of diastereomers 4*S* and 4*R*. Both diastereomers, when present in the ds-oligo (nineteenmer) structure, cause a decrease in melting temperature of 15 °C [30]. Therefore, the (*R*/*S*)-Sp formation in the ds-oligo structure can affect the charge transfer process, especially when it constitutes part of the clustered DNA damage, together with its precursor ^OXO^dG. It should be pointed out that apart from two diastereomeric forms, spirodi(iminohydantoin) can adopt a *syn* or *anti* conformation (Figure 2). The four ds-oligonucleotides above were taken into theoretical consideration, as presented in Table 1.

### 2.1. Analysis of ds-Oligos Spatial Geometries

The spatial geometries of the discussed short ds-DNA were optimized using ONIOM methodology at the M02-2X/D95**||M06-2X/STO-3G level of theory in the aqueous phase using the conductor-like polarizable continuum model (CPCM). The details are presented in the Section 3.1 and Section 3.2. Extraction of spirodi(imonohydantoin) from the mentioned oligos revealed changes not only in the mutual 2-deoxyribose/base orientation but also in the sugar puckering [31]. The following furanose ring conformation was assigned: *syn* (*S*)-Sp: ^2^_1_T; *anti* (*S*)-Sp: ^0^_1_T; *syn* (*R*)-Sp: ^3^_4_T; *anti* (*R*)-Sp: ^2^_1_T (Figure 2). The above indicates that the spirodi(iminohyantoin) rotation around the glycosidic bond (C_1_’-N_9_) forces ribose conformation changes from S to N with subsequent rotation barrier increases in the case of the (R)-Sp diastereomer. The difference (absolute value) between the *syn* and *anti* conformers of (*R*)-Sp was 1.31 kcal, while for (*S*)-Sp, it was noted as more than twice as low, i.e., 0.61kcal. This corresponds well with the stability of the assigned conformers as follows: *syn*(*S*)-Sp > *anti*(*S*)-Sp > *anti*(*R*)-Sp > *syn*(*R*)-Sp. As shown in Figure 1 and mentioned above, Sp is the yield from dG or ^OXO^dG. However, if spirodi(iminohydantoin) formation takes place in the double helix structure, its electronic properties and spatial structure should be discussed in the context of a base pair with 2’-deoxycytidine. As shown in previous research, the structure of isolated DNA lesions is nothing like that which is found in ds-DNA [32]. Here, the influence of four different forms (Figure 2) of Sp on the structure of the ds-oligo (presented in Table 1) has been investigated. The stability of the double helix depends on hydrogen bond energy and the stacking interaction between proximal base pairs, in addition to the first solvation layer [33]. All this directly influences charge transfer through the ds-oligo and the mutational potential in the replication step [34,35].

**Figure 3:**
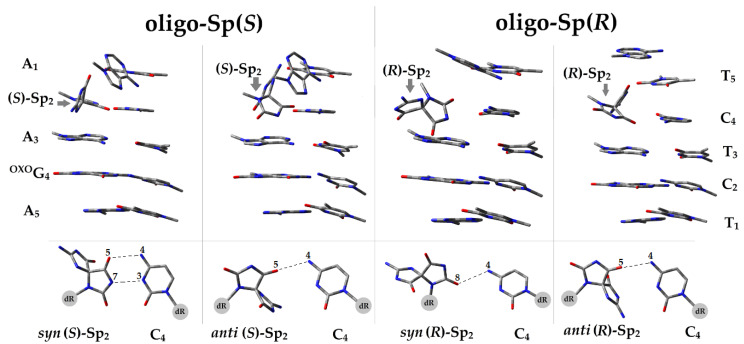
Spatial geometry of optimized *ds*-oligo presented in Table 1, d[A_1_Sp_2_A_3_^OXO^G_4_A_5_] * d[T_5_C_4_T_3_C_2_T_1_] containing different diastereomers and conformers of spirodi(iminohydantoin), as well as a representation of the hydrogen bond of Sp_2_C_4_ base pairs.

As shown in Figure 3, the presence of Sp forces significant structural changes in the double helix depending on its confirmation and diastereomeric form. A careful analysis of the hydrogen bond energies (*E*_HB_) revealed significant decreases in the anti(*R*)-Sp_2_C_4_ base pair. The negative effect was propagated over the A_1_T_5_ moiety, located on the 5’-end of the discussed ds-oligos. In both cases, the E_HB_ was reduced to 3.2 kcal. Furthermore, when the *anti*(*R*)-Sp_2_ is present, it can form a non-canonical hydrogen bond with the T_5_ moiety of the complementary strand between atoms N1–N3 (2.86Å) and N2–O5 (2.86 Å) with 10.22 kcal of *E*_HB_. The above caused the distance between the nitrogen atom N9 of A_3_ and N9 of A_1_ moieties to increase by up to 9.11 Å, with an analogous distance between bases A_5_ and A_3_ of 7,5 Å. Additionally, a reduction of the complementary base pair A_1_T_5_‘s energy of 0.12 kcal was noted. It should be pointed out that the complementary pyrimidine strand was almost unaffected. Therefore, it can be postulated that the presence of *anti*(*R*)-Sp in the ds-DNA structure frees up the adjacent 5′-end base, which can lead to additional nucleotide insertion during the replication process, as shown in Figure 3. The situation was similar when *anti*(*S*)-Sp or *syn*(*R*)-Sp is present. The hydrogen bond energy between spirodi(iminohydantoin) and complementary cytidine were found to be as follows: 2.44 and 3.25 kcal, respectively. However, in both cases, the *E*_HB_ of the A_1_T_5_ base pair was at the same level as that calculated for the A_5_T_1_ moiety, i.e., 10.07 kcal. It should be pointed out that *syn*(*R*)-Sp leads to a modified base flip-out from the double helix, leaving the geometry of the remaining base pairs almost unaffected. As mentioned above, *anti*(*S*)-Sp causes *E*_HB_ to decrease (2.44 kcal). The rotation of spirodi(iminohydantoin) around the glycosidic bond to a *syn* conformation causes *E*_HB_ increases of up to 10.95 kcal, which is at the level of A::T base pair HB energy (Table 2). Figure 3 shows a formed non-canonical HB between *syn*(*S*)-Sp_2_ and C_4_, i.e., O5–N4 (2.93Å) and N7–N3 (2.91Å). At this point, it should be pointed out that the part of ds-oligo linked to the Sp 3’-end was almost unaffected in all the discussed cases. This observation together with the higher *syn*(*S*)-Sp stability indicates that the main destabilizing double helix effect is derived from the structural changes forced in the 5’-end direction of spirodi(iminohydantoin).

The stability of the double helix also depends on the π-stacking interaction between the neighboring base pairs, as shown in Table 2. The above is also crucial for the charge transfer process through ds-DNA. Because of the strong structural differences between canonical 2′-deoxguanosine and spirodi(iminohydantoin), differences in stacking energy (*E*_ST_) should be observed, especially when different forms of Sp are considered. The average *E*_ST_ on BP dimers of an analogous ds-pentamer, i.e., d[A^OXO^GA^OXO^GA] * [TCTCT], was noted as 15.5 kcal (sd = 0.8). Here, in all the discussed ds-oligos, the stacking energy of the [A^OXO^GA] * [TCT] moiety was found at the same level, irrespective of the form of Sp (16.26 kcal, sd = 0.9). The situation becomes different when the spirodi(iminohydantoin) was taken into consideration. When Sp adopts the *syn* conformation, irrespective of the diastereomeric 4*R* or 4*S* form, *E*_ST_ decreases of up to ~10 kcal were observed for the A_1_T_5_|Sp_2_C_4_ interaction (Table 2). No such effect was noted for *anti* conformers. Instead, a reduction in *E*_ST_ was noted for Sp_2_C_4_|A_3_T_3_ base pair dimers. However, a greater negative effect was exerted by the presence of the *anti*(*R*)-Sp moiety (10.95 kcal) than the opposite diastereomer (13.68 kcal). The above is probably the result of A_3_ A_1_ distance elongation and A_1_T_5_HBs de-pairing, which is shown in Figure 3 and has been discussed previously. From the results presented above, it can be concluded that the hydrogen bonds and stacking energies around the ^OXO^G location are not affected by the second CDL, i.e., Sp, regardless of its conformation and stereochemistry. Furthermore, both spirodi(iminohydantoin) diastereomers, when adopting the *anti* conformation, exert a negative effect towards the 3’-end direction of the purine strand. Sp rotation around the glycosidic bond and *syn* conformation formation leads to a reduction in *E*_ST_ towards the opposite5’-end (Table 2).

### 2.2. The Electronic Properties of ds-Oligonucleotides

As shown by Sugyama, the Gs island (GG, GGG, etc.) is predisposed to adopt the lowest ionization potential and become a sink for the radical cation migrating through the double helix (hole, electron–hole migration) [36]. The above has been also confirmed by Gurea and Voityuk’s systematic studies of nucleobase trimmers [37,38]. Moreover, Sevillas’ recent studies have shown the significant influence of ^OXO^dG on charge distribution. Because of this and the influence of spirodi(iminohydantoin) on structure and hydrogen bond/stacking energies, the electronic properties of ds-oligo, as presented in Table 1, were considered. For these proposals, only the base pair ladder was investigated at the M06—2x/6—31++G** level of theory in the aqueous phase, which was the limit of applied computational power for such an extended system. Additionally, equilibrated (EQ) and non-equilibrated (NE) solvent–solute interaction was also implemented. The one-electron oxidizing process causes radical cation formation. The calculated values of vertical ionization potential (VIP) in NE and EQ modes revealed no difference between ds-oligos containing the (*R*)-Sp or (*S*)-Sp diastereomers. However, in each case, the presence of an *anti* conformer leads to slightly greater VIP^NE/EQ^ and adiabatic ionization potential decreases in comparison to *syn*, as shown in Table 3. The situation becomes more complicated when the excess electron appears in the ds-oligo. The vertical electron affinity (VEA) values calculated in the solvent–solute non-equilibrated and equilibrated modes were found at almost the same level for all analyzed ds-oligos, with differences of 0.07 eV and 0.01 eV, respectively. After structure relaxation and adiabatic anion state formation, a higher adiabatic electron affinity (AEA) was noted in the following order: oligo-(S)Sp*^ANTI^*~oligo-(R)Sp*^ANTI^* > oligo-(R)Sp*^SYN^* > oligo-(S)Sp*^SYN^*. It should be pointed out that the appearance of *ant*i conformer 4*S* spirodi(iminohydantoin) in the ds-DNA causes significant AEA decreases of up to −1.34 eV. The above can be directly derived from the nature of the formed base pair between Sp_2_ and C_4_, which is different in comparison to others (see Figure 3 and Table 3).

### 2.3. The Electronic Properties of Isolated Base Pair

Due to the lack of major differences in global electronic properties between the discussed ds-oligonucleotides, single base pairs isolated from the double helix were taken into consideration. However, the non-equilibrated solvent–solute interaction was omitted. The above is the result of ds-oligo solvation shell mode, with no water molecules present between the stacked base pairs. The preferred place for radical cation formation within ds-DNA, after one-electron oxidizing, can be estimated by analyzing the electronic properties of isolated base pairs. All parameters were calculated at the M06—2X/6—31++G** level of theory in the CPCM mode. The calculated VIP and AIP values were found at the same level for the analyzed base pairs, except for ^OXO^G_4_C_2_, as shown in Table 4. However, in all the considered ds-oligo cases (Table 1), ^OXO^G_4_C_2_ adopted the same, lowest VIP and AIP values, i.e., 5.9 eV, 5.5 eV, respectively. This would indicate that the base pair formed by 7,8-dihydro-8-oxo-2’-deoxyguanosine and dC is the preferred point for the radical cation to settle, regardless of the presence of other lesions in the clustered DNA damage structure. With all of the above in mind, it can be postulated that the electron hole migrates through a double helix containing different forms of spirodi(iminohydantoin) in an almost unaffected manner until it reaches the place with the lowest ionization potential, i.e., the ^OXO^GC moiety. On the other side of the charge transfer, the excess electron appearing in the system leads initially to vertical anion radical formation with its subsequent rearmament to adiabatic form. The above can be characterized by vertical and adiabatic electron affinity denoted as VEA and AEA, respectively. Contrary to the results obtained for the radical cation, the base pair which shows the lowest VEA is different from the base pair which shows the lowest AEA. The above distribution depends on the forms of spirodi(iminohydantoin). As presented in Table 4, the lowest VEA and AEA were noted for the following base pairs: oligo-Sp(*R*)*^ANTI^*:^OXO^G_4_C_2_ (in both cases), oligo-Sp(*R*)*^ANTI^*:^OXO^G_4_C_2_ and A_5_T_1_, oligo-Sp(*S*)*^ANTI^*:A_1_T_5_ and ^OXO^G_4_C_2_, oligo-Sp(*S*)*^SYN^*:^OXO^G_4_C_2_and A_1_T_5_, respectively. In addition, notable differences between the VEA and AEA of the base pair were only observed in the case of the base pair for which the lowest AEA was noted, apart from oligo-Sp(*R*)*^SYN^*, in which the spirodi(iminohydantoin) was flipped out from the double helix structure (Figure 3). The VEA and AEA of *syn*(*R*)-Sp_2_C_4_ were assigned as −1.14 and 1.49 eV, respectively, while for the others this difference was predominately below 0.04eV (in each ds-oligo case).

### 2.4. The Charge and Spin Distribution

**Figure 4:**
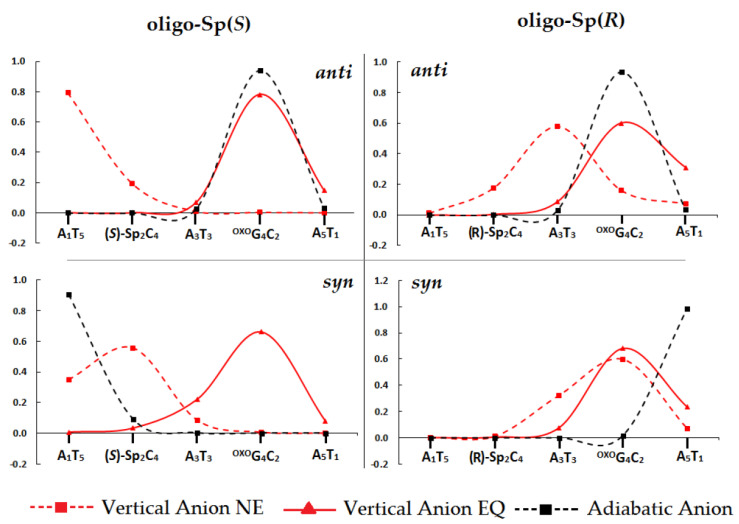
Spin distribution within oligo-Sp(*S*)^AN*TI*^, oligo-Sp(*S*)*^SYN^*, oligo-Sp(*R*)*^ANTI^* and oligo-Sp(*R*)*^SYN^* calculated at the M062x/6-31++G** level of theory in the condensed phase, the base pairs ladder being taken into consideration. NE: non-equilibrated solvent–solute interaction, EQ: equilibrated solvent–solute interaction. The raw data of charge and spin distribution are given in Table S4 of the Supplementary Materials.

The results discussed above indicate that differences in the spin and charge distribution between the ds-oligos should be observed. As mentioned previously, all energies of vertical and adiabatic positively and negatively charged ds-oligo forms were investigated at the M06—2X/6—31++G** level of theory in the aqueous phase. Here, for charge and spin distribution, the Hirshfeld methodology was used [39]. The loss of the electron leads to vertical radical cation state formation. The spin distribution of positively charged ds-oligonucleotides shows that a one-electron oxidizing process occurs almost exclusively on the ^OXO^G_4_C_2_ moiety, i.e., more than 90% (Table S4) in all of the discussed stages, i.e., vertical cation calculated in non-equilibrated and equilibrated solvent–solute interaction modes, as well as after macromolecule structure relaxation (adiabatic cation). The above indicates that spirodi(iminohydantoin), irrespective of stereochemistry and conformation, is not a hole electron competitor for ^OXO^G even if it forms part of a CDL. This result is in strong agreement with previously determined VIPs and AIPs of base pairs isolated from ds-oligo (Table 1 and Table 4). The excesses electron appearing in the macromolecule structure leads to a different negative charge and spin distribution depending on the Sp C4 carbon chirality and base-sugar spatial arrangement, as presented in Figure 4.

As previously, analyses were performed for the vertical anion (VA) radical state, and adiabatic (AA) on the NE and EQ solvent–solute mode were also considered (see Table S4). The strong influence of Sp on the double helix spatial structure (Figure 3) was also indirectly visible in the spin distribution. A higher density was noted for the VA^NE^ state as follows: oligo-Sp(*S*)^ANTI^—A_1_T_5_ (79%); oligo-Sp(*R*)^ANTI^—A_3_T_3_ (58%); oligo-Sp(*S*)^SYN^—*syn*(*S*)-Sp_2_C_4_ (56%); oligo-Sp(*R*)^SYN^—^OXO^G_4_C_2_ (60%). The solvent–solute interaction equilibration, i.e., VA^EQ^ state achievement, causes a spin shift: oligo-Sp(*S*)^ANTI^—^OXO^G_4_C_2_ (78%); oligo-Sp(*R*)^ANTI^—^OXO^G_4_C_2_ (60%); oligo-Sp(*S*)^SYN^—^OXO^G_4_C_2_ (66%); oligo-Sp(*R*)^SYN^—^OXO^G_4_C_2_ (68%). The structural “relaxation” after the appearance of an excess electron leads to adiabatic radical anion formation (the geometry of the anion has been optimized to the ground state). An analysis of the AA state showed that a higher density of spin (unpaired electron) was on the ^OXO^G_4_C_2_ of the oligo-Sp(*S*)^ANTI^ and oligo-Sp(*R*)^ANTI^ at 94% and 92% respectively. The rotation of the Sp moiety around the glycosidic bond and *syn* conformation adoption caused the excess electron to settle at 91% on A_1_T_5_ of oligo-Sp(*S*)^SYN^ and at 98% on the opposite end of the ds-DNA, i.e., A_5_T_1_ of oligo-Sp(*R*)^SYN^. All the above indicates that the spirodi(iminohydantoin) exerts a significant influence on electron distribution even if the ^OXO^dG is present in the clustered DNA damage structure. Furthermore, this influence is directly connected to its diastereomeric (4*R* or 4*S*) forms and adopted conformation, *anti* or *syn* (cf. Figure 3 and Figure 4). No influence at all was noted in the case of radical cation propagation. The positive charge and accompanying unpaired electron (spin) were always located at the ^OXO^G_4_C_2_ base pair of all ds-oligos. This is in strong agreement with previous experimental and theoretical studies, which predicted that ^OXO^G becomes a radical cation sink if appearing in a double helix structure. Therefore, ^OXO^G can undergo further rearrangement with other DNA lesions and protect the proximal parts of the double helix against oxidation. However, during this process, the mutagenicity of subsequent products of ^OXO^G increases from 3% to as much as 100%, as was found for spirodi(iminohdantoin). Therefore, the influence of clustered DNA damage, the level of which increases during radiotherapy, on the DNA repair process is important in terms of genome stability of normal cells and the effectiveness of radio/chemo anticancer therapy.

### 2.5. The DNA Charge Transfer

Charge transfer through a double helix can be discussed in terms of excess electrons or hole mode [40]. Knowledge of the above is important for understanding how proteins involved in genetic material repair and replication communicate with each other. Barton et al.postulated that glycosylases such as MutY and ExoIII can scan the *Escherichia coli* (*E. coli*) genome and detect lesions (e.g., ^OXO^GA) in less than 10 min by electron transfer between these two proteins [41]. In a single *E.coli* cell, around 30 copies of MutY exist [42]. According to the common supposition that charge transfer depends on mutual base pair geometry*,* there has been much interest in investigating the influence of CDL on charge transfer. The above is important for effective and safe radiotherapy as the number of CDLs in a cell increase, which ultimately affects single lesion repairs [19]. Although hydrolytic glycosylase activity is well understood, our understanding of DNA damage detection in the vast majority of nucleosides is as yet vague. Lesion recognition is important because glycosylases initiate the cascade of further proteins involved in BER machinery and protect the cell against unwanted mutation.

The charge transfer can be perceived in three categories: single-step tunneling, random-walk multistep, and polaron-like hopping, both in excess electron and electron hole modes [43,44]. The structure of the double helix enables their migration over a long distance—at least 200 Å—from its induction point, according to an incoherent mechanism [45]. On the other hand, a single-step super exchange mechanism can be observed for the distance of a few base pairs.

According to Marcus’ theory, the charge transfer depends on the following factors: *k*_HT_ (rate constant, s^−1^), ΔG (driving force, eV), energies *λ* (reorganization, eV) and *E*_a_ (activation, eV), as well as *V*_12_ (electron coupling, eV) [46,47,48]. All the above parameters have focused on *k*_HT_ (Equation (1)),

(1) $$k_{\mathrm{HT}}=\frac{4\pi {\left|V_{12}\right|}^{2}}{h}\times \sqrt{\frac{1}{4\pi \lambda k_{b}T}}\times exp\left(\frac{-{\left(∆G+\lambda \right)}^{2}}{4\pi k_{b}T}\right)$$

while *E*_a_ is described by Equation (2) (where: *k*_b_ is the Boltzmann constant, *h* is the Planck constant, and *T* is the temperature in [K]).

(2) $$E_{a}=\frac{\lambda }{4}\times {\left(1+\frac{∆G}{\lambda }\right)}^{2}$$

In these studies, electron coupling (*V*_12_) was calculated according to the Generalized Mulliken–Hush method (GMH) and described by Equation (3) [49]. (Δ*E*_12_-vertical excitation energy (eV), *μ*_1_ − *μ*_2_ is the difference between the ground and first excited dipole moment in Debyes, while *μ*_12_ is the-transition dipole moment).

(3) $$V_{12}=\frac{∆E_{12}\left|\mu_{12}\right|}{{\left(\mu_{1}-\mu_{2}\right)}^{2}+4\mu_{12}^{2}}$$

The analysis of the mentioned parameters presented in Table 5 shows that the conformer *syn* of (4*S*) spirodi(iminohydantoin) almost completely inhibits the Sp_2_C_4_→A_3_T_3_ hole transfer. A significantly high value of *E*_a_ was found, which results in a small *k*_HT_ = 3.5 × 10^−100^ value. For the other ds-oligos, this migration was characterized by a small value of *E*_a_ and *k*_HT_ in a range of 10^13^ to 10^14^. A significant slowing down of the hole migration process was also observed for the radical cation transfer from Sp_2_C_4_→^O^G_4_C_2_ in the case of oligo-Sp(*R*)*^ANTI^*, oligo-Sp(*S*)*^SYN^k*_HT_ = 3.6 × 10^−19^, *k*_HT_ = 7.4 × 10^−23^. As can be expected, in every case, the hole transfer towards the ^OXO^G_4_C_4_ base pair was found, provided *k*_HT_ was assigned in the range of 10^8^–10^12^s^−1^. This is in strong agreement with previous results of charge/spin distribution as well as the electronic properties of single base pairs. Because the base pairs were isolated from the ds-oligo in neutral, cationic and anionic adiabatic forms, in some cases λ adopted a small negative value close to zero (Table 5), which finally resulted in a negative *E*_a_. These non-physical values could indicate that the charge transfer through these base pairs takes place without any resistance—no nucleus rearmament is required. The above supports the previous observation that charge can migrate through ds-DNA over a long distance.

There is a significant difference between this study, in which relatively short ds-oligo fragments have been discussed, and previous studies, in which only the simple base pair dimer was considered [50,51]. The appearance of the migrated electron in the ds-oligo causes fewer visible changes in rearrangement energy (λ), which indicates a smaller structural influence than was noticed for the radical cation (Table 5). The highest λ values were calculated for base pairs between which a charge transfer occurred with a final charge/spin density rearrangement (Table 5 and Figure 4).

The above was found as follows: A_1_T_5_←Sp_2_C_4_ and Sp_2_C_4_←A_3_T_3_ for oligo-Sp(*S*)*^SYN^*, ^O^G_4_C_2_→A_5_T_1_ of oligo-Sp(*R*) *^SYN^*, A_3_T_3_→^O^G_4_C_2_ of oligo-Sp(*S*) *^ANTI^* and A_3_T_3_→^O^G_4_C_2_, ^O^G_4_C_2_←A_5_T_1_ for oligo-Sp(*R*) *^ANTI^*. In all the discussed cases, the rate constant was noted at a level of 10^12^–10^13^s^−1^. Conversely, only in the case of A_1_T_5_←X_2_C_4_ of oligo-Sp(*R*)*^ANTI^* was a significant electron transfer inhibition observed, i.e.: *k*_HT_ = 2.3 × 10^−9^. The above could be the result of the structural change forced by the presence of *anti*(*R*)-Sp. The distance between A_1_T_5_ A_3_T_3_ increases by up to 9 Å, with A_1_ flipping out from the base pair structure (Figure 4). Finally, comparing the hole and electron transfer rate constants (Table 5) revealed that the *k*_HT_ of electron transfer is a few orders of magnitude higher than that obtained for the radical cation transfer, which is in strong agreement with previous results [52].

### 2.6. Final Remarks and the Outlook for the Future

The research findings presented in this study could contribute valuable knowledge towards ensuring the safety of future generations. Mankind’s enduring desire to discover worlds beyond Earth requires a study of astronaut safety protocols and further research into nutrition, among other factors. The heightened levels of cosmic and solar radiation beyond the protective magnetosphere of our planet pose a significant challenge to any prospective colonization of the Moon and Mars. Additionally, this research has important implications with regard to our increased life expectancy: DNA damage repair machineries slow down and deteriorate with age. As our longevity increases, inevitably, so too does the number of incidents of cancer. Given so-called patient-friendly radiotherapy treatment for killing cancer cells uses ionization radiation that can also damage healthy cells, it is vital that further research be carried out. The data presented in this study could be promising in terms of facilitating a more effective and controlled dose delivery to reduce undesired damage to the healthy tissue surrounding a tumor during radiotherapy.

Spirodi(iminohydantoin) is formed of specific flexible nucleoside analogues arranged in a definite conformation, and has helix-distorting features. Furthermore, it influences π-stacking between neighboring base pairs, all of which means that its presence in the genome affects the charge transfer, as shown. The above can be manifested by the disordered activity of [4Fe–4S] proteins which exploit a charge transfer to either recognize and repair other DNA damage or to replicate genetic information. The crucial proteins involved in the DNA lesion recognition process as well as genetic material replication contain [4Fe–4S] clusters. Therefore, different Sp conformers can indirectly lead to the collapse of DDR and replication machineries, also giving rise to aging, carcinogenesis and cell death.

Therefore, it is imperative to clarify the role of Sp in both diastereomeric forms in the “DNA recognition and repair machinery” of local multiple damaged sites composed of different kinds of lesions. Future research could address the question of Sp’s influence on the CDL recognition and repair process by proteins containing [4Fe-4S] cluster. Additionally, the experimental investigation of Sp’s influence on CT through the double helix induced by ionization radiation should shed valuable light on whether Sp plays an insulating or transmitting role during radical cation or electron migration and whether it plays a significant role in DNA damage formation in the distal part of the double helix.

## 3. Materials and Methods

### 3.1. Computation Methodology of ONIOM Studies

The initial neutral, cation and anion structure form optimizations of the ds-oligos presented in Table 1 were performed using ONIOM (our own n-layered integrated molecular orbital and molecular mechanics) methodology [53]. For this study, the previously described computation methodology was applied [32]. The structures of all ds-pentamers were divided into nucleobases (high-level) M06—2X/D95**, and sugar–phosphate backbone (low-level) M06—2X/STO—3G. All calculations were performed in the aqueous phase. Because of the complexity of the system, the negative charges of all the phosphate groups were neutralized by the addition of a proton, instead of counterions [54,55,56]. The obtained ds-pentamers were subsequently converted into a base pair ladder, which was further used for electronic property energy calculations, in line with previous studies [32].

### 3.2. Computation Methodology of DFT Study [32]

All energy calculations were performed in the aqueous phase using density-functional theory (DFT) using the M06—2X functional with a 6—31++G** basis set [57,58]. The transition dipole moment of excited states and the single point calculation at the M06—2X/6—31++G** level of theory were performed using time-dependent density-functional theory (TDDFT) [59,60]. The solvent effect was described for an aqueous medium, applying Tomasi’s polarized continuum model (CPCM) [61]. The solvent–solute interaction was looked at in two modes, i.e., non-equilibrium (NE) and equilibrated (EQ) [62]. For all the discussed ds-oligos, charge and spin analyses were carried out using Hirshfeld theory [39]. Electron coupling was calculated according to the Generalized Mulliken–Hush methodology [49]. The electronic properties, i.e., adiabatic ionization potential (AIP), adiabatic electron affinity (AEA), vertical ionization potential (VIP), and vertical electron affinity (VEA), were calculated as previously described [63]. All calculations were performed in the aqueous phase on the Gaussian G16 (version C.01) software package [64].

## 4. Conclusions

The genome is continuously exposed to harmful factors such as ionization radiation, reactive oxygen/nitrogen species, etc. Of all the five nucleobases, guanine has the lowest ionization potential, and is thus most easily oxidized to 7,8-dihydro-8-oxo-2’-deoxyguanosine. Such DNA lesions have been intensively studied for many years and assigned as the most common lesion, which is readily used as a marker of pathogenic processes. It has been established that the mutagenic potential of ^OXO^G is at a level of 3%. In favorable conditions, with a pH of >5.7, it can be converted to spirodi(iminohydantoin), which is highly mutagenic.

In this study, the electronic properties and the influence on charge transfer through ds-DNA of Sp were investigated. Sp can exist in the genome in two diastereomeric forms, 4*R* and 4*S,* which adopt a *syn* or *anti* conformation. With this in mind, four ds-pentamers (shown in Table 1) containing CDLs were investigated.

- A structural analysis revealed the following stabilities of base pairs formed by spirodi(iminohydantoin): *syn*(4*S*)-Sp > *anti*(4*S*)-Sp > *anti*(4*R*)*-*Sp > *syn*(4*R*)*-*Sp. Additionally, the presence of an *anti* conformer of (4*R*)*-*Sp causes purine strand elongation with subsequent hydrogen bond elimination in the 5’-end adjected base pair.
- The electronic properties of base pairs isolated from ds-DNA show that in all cases, ^OXO^GC adopted the lowest ionization potential, irrespective of the spirodi(iminohydantoin) form. Conversely, a higher adiabatic electron affinity was found for ^OXO^G_4_C_2_ of oligo-(*R*)Sp^ANTI^ and oligo-(*S*)Sp^ANTI^ than for oligo-(*R*)Sp^SYN^ and oligo-(*S*)Sp^SYN^ for A_5_T_1_ and A_1_T_5_.
- The above strongly concurs with the charge and spin distribution analysis: for all the ds-oligo radical cations, a higher spin density was noted for the ^OXO^GC moiety, irrespective of the Sp form. For the ds-oligo radical anion, the localization of an unpaired electron is strictly dependent on Sp conformation and its diastereomeric form. A higher density was found on ^OXO^GC of oligo-Sp(*R*)^ANTI^ and oligo-Sp(*S*)^ANTI^, while in the case of oligo-Sp(*S*)^SYN^ and oligo-Sp(*R*)^SYN^, a higher density was located on the opposite ends of the double helix, i.e., A_1_T_5_ and A_5_T_1_ respectively.
- The influence of spirodi(iminohydantoin) on charge transfer was investigated according to Marcus’ theory. In most cases, Sp did not become a significant dam for hole migration, except for anti (4*S*)-Sp, which affected the transfer towards the base pair adjected to its 3’-end (A_3_T_3_). Furthermore, the radical cation transfer towards the ^OXO^G_4_C_2_ base pair from proximal base pairs was found at a 10^7^–10^12^ s^−1^ level of rate constant. The situation was different in the case of excess electron migration and a significant decrease in the charge transfer rate was noted for A_1_T_5_←(4*R*)*-*Sp_2_C_4_ of oligo-Sp(*R*)^ANTI^ and ^OXO^G_4_C_2_←A_5_T_1_oligo-Sp(*S*)^ANTI^.

In conclusion, DNA damage to spirodi(iminohydantoin), especially when becoming part of clustered DNA damage, can affect the efficiency of the lesion recognition and repair process because it exerts a strong influence on the electron transfer process. This can in turn lead to an acceleration of undesirable/dangerous processes such as carcinogenesis or aging. However, from the anticancer radio-/chemo- or combined therapy point of view, a slowing down of repair machinery can lead to increased effectiveness. Therefore, the influence of clustered damage on charge transfer and subsequently on single damage recognition by glycosylases warrants future investigation.

## Figures and Tables

**Table 1:** The sequence and notation of *ds*-oligonucleotides used for these studies.

Ds-Oligonucleotide Sequence	Notation
d[A_1_(*S*)Sp_2_^S*YN*^A_3_^OXO^G_4_A_5_] * d[T_5_C_4_T_3_C_2_T_1_]	*oligo-*(*S*)Sp^SYN^
d[A_1_(*S*)Sp_2_*^ANTI^*A_3_^OXO^G_4_A_5_] * d[T_5_C_4_T_3_C_2_T_1_]	*oligo-*(*S*)Sp^ANTI^
d[A_1_(*R*)Sp_2_*^SYN^*A_3_^OXO^G_4_A_5_] * d[T_5_C_4_T_3_C_2_T_1_]	*oligo-*(*R*)Sp^SYN^
d[A_1_(*R*)Sp_2_*^ANTI^*A_3_^OXO^G_4_A_5_] * d[T_5_C_4_T_3_C_2_T_1_]	*oligo-*(*R*)Sp^ANTI^

**Table 2:** Stacking and hydrogen bond energies calculated at the M06—2x/6—31++G** level theory in the condensed phase as well as (**A**) stacking interaction and (**B**) a graphical representation of the HBs. The raw data are given in Tables S1 and S2 of the Supplementary Materials.

System	Oligo-Sp(*S*)		Oligo-Sp(*R*)		System	Oligo-Sp(*S*)		Oligo-Sp(*R*)	
	*Anti*	*Syn*	*Anti*	*Syn*		*Anti*	*Syn*	*Anti*	*Syn*
BP Dimer	Stacking Energy				Base Pair	Hydrogen Bond Energy			
A_1_T_5_|Sp_2_C_4_	15.32	10.30	17.77	9.89	A_1_T_5_	10.07	9.16	3.36	10.62
Sp_2_C_4_|A_3_T_3_	13.68	17.19	10.95	14.97	Sp_2_C_4_	2.44	10.95	3.17	3.25
A_3_T_3_|^O^G_4_C_2_	16.55	15.81	17.21	17.75	A_3_T_3_	9.85	10.88	10.77	9.22
^O^G_4_C_2_|A_5_T_1_	16.25	15.82	15.22	15.46	^OXO^G_4_C_2_	18.13	18.08	17.79	17.97
					A_5_T_1_	10.60	10.63	10.63	10.66
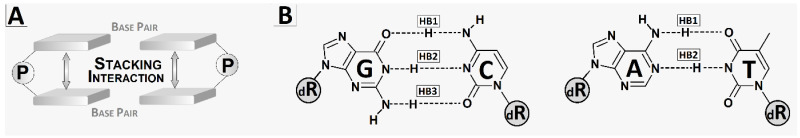									

**Table 3:** The electronic properties of ds-oligonucleotides presented in Table 1 at the M06—2X/6—31++G** level of theory in the aqueous phase. All values are given in eV.

Conformer	VIP^NE^	VIP^EQ^	AIP	VEA^NE^	VEA^EQ^	AEA
oligo—Sp(*S*)						
*anti*	6.35	5.88	5.37	−0.68	−1.44	−1.97
*syn*	6.66	5.90	5.48	−0.67	−1.34	−1.34
**oligo—Sp(*R*)**						
*anti*	6.39	5.81	5.38	−0.62	−1.37	−1.94
*syn*	6.64	5.88	5.43	−0.69	−1.38	−1.87

**Table 4:** The electronic properties, in [eV], of base pairs isolated from discussed ds-oligos: Vertical (VIP), Adiabatic Ionization Potential (AIP) and Vertical (VEA), Adiabatic (Electron Affinity AEA) calculated at the M062x/6—31++G** level of theory in the aqueous phase. The raw data are given in the Supplementary Materials, Table S3.

Electronic Properties In [eV]								
oligo-Sp(*R*)					oligo-Sp(*S*)			
Base Pair	*anti*		*syn*		*anti*		*syn*	
	VIP	AIP	VIP	AIP	VIP	AIP	VIP	AIP
A_1_T_5_	6.76	6.74	6.75	6.76	6.55	6.52	6.62	6.65
Sp_2_C_4_	7.66	7.64	6.93	6.91	6.91	6.90	7.69	7.72
A_3_T_3_	6.62	6.61	6.84	6.56	6.80	6.50	6.64	6.61
^OXO^G_4_C_2_	5.93	5.53	5.91	5.54	5.91	5.56	5.90	5.56
A_5_T_1_	6.73	6.68	6.71	6.67	6.72	6.68	6.72	6.67
**oligo-Sp(*R*)**					**oligo-Sp(*S*)**			
**Base Pair**	***anti***		***syn***		***anti***		***syn***	
	**VEA**	**AEA**	**VEA**	**AEA**	**VEA**	**AEA**	**VEA**	**AEA**
A_1_T_5_	−1.51	−1.51	−1.40	−1.39	−1.57	−1.60	−1.42	−1.85
Sp_2_C_4_	−1.32	−1.33	−1.33	−1.32	−1.29	−1.34	−1.14	−1.49
A_3_T_3_	−1.44	−1.34	−1.43	−1.42	−1.41	−1.42	−1.30	−1.44
^OXO^G_4_C_2_	−1.52	−1.97	−1.53	−1.53	−1.53	−1.96	−1.50	−1.48
A_5_T_1_	−1.43	−1.41	−1.43	−1.83	−1.42	−1.38	−1.44	−1.44

**Table 5:** Charge transfer parameters: λ-reorganization energy, ΔG-driving force, *E*_a_-activation energy, *V*_12_-electron coupling, and *k*_HT_-charge rate constant of permissible transfers between base pairs of ds-oligo presented in Table 1, calculated at the M06—2X/6—31++G** level of theory in the aqueous phase and given in [eV]. Arrows indicate the direction of charge migration. The raw data are given in Tables S5 and S6 of the Supplementary Materials.

Excess-Electron Transfer											
System	λ	ΔG	*E*_a_	*V*_12_	*k_HT_*	System	λ	ΔG	*E*_a_	*V*_12_	*k_HT_*
*X:syn (S)-Sp*	oligo-Sp(*S*)*^SYN^*					*X:syn (R)-Sp*	oligo-Sp(*R*)*^SYN^*				
A_1_T_5_←X_2_C_4_	0.51	−0.36	0.01	0.11	1.9 × 10^14^	A_1_T_5_←X_2_C_4_	0.03	−0.07	0.01	0.01	6.6 × 10^12^
X_2_C_4_←A_3_T_3_	0.34	−0.05	0.06	0.07	3.4 × 10^13^	X_2_C_4_→A_3_T_3_	0.03	−0.09	0.04	0.02	6.6 × 10^12^
A_3_T_3_→^OXO^G_4_C_2_	−0.02	−0.04	−0.04	0.01	N.D	A_3_T_3_→^OXO^G_4_C_2_	0.01	−0.11	0.36	0.03	1.2 × 10^8^
^OXO^G_4_C_2_←A_5_T_1_	−0.02	−0.04	−0.05	0.05	N.D	^OXO^G_4_C_2_→A_5_T_1_	0.47	−0.30	0.01	0.05	4.2 × 10^13^
A_1_T_5_←A_3_T_3_	0.43	−0.41	0.00	0.07	1.3 × 10^14^	A_1_T_5_→A_3_T_3_	0.01	−0.03	0.03	0.06	2.2 × 10^14^
X_2_C_4_←^OXO^G_4_C_2_	0.30	−0.01	0.07	0.06	4.0 × 10^12^	X_2_C_4_→^OXO^G_4_C_2_	0.04	−0.21	0.18	0.06	3.3 × 10^11^
A_3_T_3_←A_5_T_1_	0.14	−0.00	0.03	0.08	8.0 × 10^13^	A_3_T_3_→A_5_T_1_	0.41	−0.42	0.00	0.08	1.7 × 10^14^
***X:anti (S)-Sp***	**oligo-Sp(*S*)*^ANTI^***					***X:anti (R)-Sp***	**oligo-Sp(*R*)*^ANTI^***				
A_1_T_5_←X_2_C_4_	0.03	−0.27	0.41	0.05	2.9 × 10^7^	A_1_T_5_←X_2_C_4_	0.01	−0.17	1.35	0.03	2.3 × 10^−9^
X_2_C_4_→A_3_T_3_	0.00	−0.08	0.33	0.04	7.0 × 10^10^	X_2_C_4_→A_3_T_3_	−0.10	−0.01	−0.03	0.05	N.D
A_3_T_3_→^OXO^G_4_C_2_	0.44	−0.55	0.01	0.06	6.2 × 10^13^	A_3_T_3_→^OXO^G_4_C_2_	0.52	−0.63	0.01	0.02	6.3 × 10^12^
^OXO^G_4_C_2_←A_5_T_1_	0.03	−0.58	2.75	0.05	8 × 10^−33^	^OXO^G_4_C_2_←A_5_T_1_	0.47	−0.55	0.00	0.05	6.1 × 10^13^
A_1_T_5_←A_3_T_3_	0.02	−0.19	0.30	0.06	4.4 × 10^9^	A_1_T_5_←A_3_T_3_	−0.08	−0.17	−0.20	0.08	N.D
X_2_C_4_→^OXO^G_4_C_2_	0.43	−0.62	0.02	0.07	5.5 × 10^13^	X_2_C_4_→^OXO^G_4_C_2_	0.45	−0.64	0.02	0.05	3.1 × 10^13^
A_3_T_3_←A_5_T_1_	−0.02	−0.03	−0.04	0.08	N.D	A_3_T_3_→A_5_T_1_	0.06	−0.08	0.00	0.08	4.1 × 10^14^
**Hole-Electron Transfer**											
**System**	**λ**	**Δ** **G**	***E*** **_a_**	***V*** **_12_**	***k_HT_***	**System**	**λ**	**Δ** **G**	***E*** **_a_**	***V*** **_12_**	***k_HT_***
**X: syn (*S*)-Sp**	**oligo-Sp(*S*)*^SYN^***					**X: syn (*R*)-Sp**	**oligo-Sp(*R*)*^SYN^***				
A_1_T_5_←X_2_C_4_	−0.02	−1.06	−19.3	0.28	N.D	A_1_T_5_←X_2_C_4_	−0.04	−0.15	−0.24	0.14	N.D
X_2_C_4_→A_3_T_3_	0.04	−1.10	6.78	0.13	3.5 × 10^−100^	X_2_C_4_→A_3_T_3_	0.26	−0.35	0.01	0.04	3.6 × 10^13^
A_3_T_3_→^OXO^G_4_C_2_	0.33	−1.05	0.40	0.38	7.3 × 10^8^	A_3_T_3_→^OXO^G_4_C_2_	0.29	−1.02	0.47	0.48	8.2 × 10^7^
^OXO^G_4_C_2_←A_5_T_1_	0.34	−1.11	0.44	0.40	1.7 × 10^12^	^OXO^G_4_C_2_←A_5_T_1_	0.37	−1.13	0.38	0.40	1.7 × 10^9^
A_1_T_5_→A_3_T_3_	0.01	−0.04	0.02	0.07	4.4 × 10^14^	A_1_T_5_→A_3_T_3_	0.29	−0.20	0.01	0.05	5.9 × 10^13^
X_2_C_4_→^OXO^G_4_C_2_	0.36	−2.16	2.26	0.61	7.4 × 10^−23^	X_2_C_4_→^OXO^G_4_C_2_	0.34	−1.38	0.77	0.56	8.7 × 10^2^
A_3_T_3_←A_5_T_1_	0.03	−0.06	0.01	0.04	1.1 × 10^14^	A_3_T_3_←A_5_T_1_	0.28	−0.11	0.03	0.07	5.4 × 10^13^
**X: anti (*S*)-Sp**	**oligo-Sp(*S*)*^ANTI^***					**X: anti (*R*)-Sp**	**oligo-Sp(*R*)*^ANTI^***				
A_1_T_5_←X_2_C_4_	0.15	−0.39	0.10	0.09	7.2 × 10^12^	A_1_T_5_←X_2_C_4_	−0.01	−0.89	−15.9	0.14	N.D
X_2_C_4_→A_3_T_3_	0.42	−0.40	0.00	0.08	1.7 × 10^14^	X_2_C_4_→A_3_T_3_	−0.02	−1.02	−11.7	0.22	N.D
A_3_T_3_→^OXO^G_4_C_2_	0.34	−0.94	0.26	0.46	2.5 × 10^11^	A_3_T_3_→^OXO^G_4_C_2_	0.41	−1.09	0.29	0.42	5.8 × 10^10^
^OXO^G_4_C_2_←A_5_T_1_	0.38	−1.12	0.36	0.41	3.8 × 10^9^	^OXO^G_4_C_2_←A_5_T_1_	0.41	−1.15	0.34	0.40	7.5 × 10^9^
A_1_T_5_→A_3_T_3_	0.32	−0.02	0.07	0.13	3.0 × 10^13^	A_1_T_5_→A_3_T_3_	0.00	−0.13	−1.08	0.05	2.1 × 10^33^
X_2_C_4_→^OXO^G_4_C_2_	0.47	−1.34	0.41	0.54	8.1 × 10^8^	X_2_C_4_→^OXO^G_4_C_2_	0.38	−2.11	1.95	0.10	3.6 × 10^−19^
A_3_T_3_←A_5_T_1_	0.28	−0.18	0.01	0.04	5.6 × 10^13^	A_3_T_3_←A_5_T_1_	0.00	−0.07	1.04	0.07	2.2 × 10^−2^

## Data Availability

All data have been attatched as Supplementary Materials file.

## Supplementary Materials

The following supporting information can be downloaded at: https://www.mdpi.com/article/10.3390/ijms24108570/s1.
