# Supplementary material for: The Influence of Spirodi(Iminohydantoin) on Charge Transfer through ds-DNA Containing 8-OXO-dG: A Theoretical Approach
Source: Int J Mol Sci. 2023 May 10;24(10):8570. doi: 10.3390/ijms24108570 (PMC10218679; doi:10.3390/ijms24108570)
Supplement: Supplementary file 1 [file ijms-24-08570-s001.zip › ijms-2325534-supplementary.pdf]

Supplementary Materials

The Influence of Spirodi(Iminohydantoin) on Charge Transfer through ds-DNA Containing 8-OXO-dG: A Theoretical Approach

Boleslaw T. Karwowski

Table S1. The energy in Hartree of base pair dimmer and adequate base pairs calculated on M06-2X/6-31++G** level of theory in aqueous phase.

BP1 // BP2	DIMER DIMMER	BP1	BP2
A_1T_5 // (*S*)$Sp^{ANTI}{}_2C_4$	-2008.864459	-921.18123	-1087.658814
(*S*)$Sp^{ANTI}{}_2C_4$ // A_3T_3	-2008.869509	-1087.658814	-921.1889
A_3T_3 // $^{OXO}G_4C_2$	-1933.693424	-921.1889	-1012.47815
$^{OXO}G_4C_2$ // A_5T_1	-1933.696264	-1012.47815	-921.192215
A_1T_5 // (*S*)$Sp^{SYN}{}_2C_4$	-2008.866677	-921.190481	-1087.659783
(*S*)$Sp^{SYN}{}_2C_4$ // A_3T_3	-2008.878962	-1087.659783	-921.191779
A_3T_3 // $^{OXO}G_4C_2$	-1933.695473	-921.191779	-1012.478496
$^{OXO}G_4C_2$ // A_5T_1	-1933.695676	-1012.478496	-921.191974
A_1T_5 // (*R*)$Sp^{ANTI}{}_2C_4$	-2008.864021	-921.180699	-1087.655006
(*R*)$Sp^{ANTI}{}_2C_4$ // A_3T_3	-2008.864346	-1087.655006	-921.191897
A_3T_3 // $^{OXO}G_4C_2$	-1933.696486	-921.191897	-1012.477165
$^{OXO}G_4C_2$ // A_5T_1	-1933.693638	-1012.477165	-921.192216
A_1T_5 // (*R*)$Sp^{SYN}{}_2C_4$	-2008.85992	-921.191237	-1087.652925
(*R*)$Sp^{SYN}{}_2C_4$ // A_3T_3	-2008.863792	-1087.652925	-921.187019
A_3T_3 // $^{OXO}G_4C_2$	-1933.692665	-921.187019	-1012.477361
$^{OXO}G_4C_2$ // A_5T_1	-1933.694296	-1012.477361	-921.192292

Table S2. The energy in Hartree of base pair and base pairs calculated on M06-2X/6-31++G** level of theory in aqueous phase.

	Base Pair	Purine	Pyrimidine
A_1T_5	-921.191698	-467.181115	-453.993557
$(S)Sp^{ANTI}{}_2C_4$	-1087.658814	-692.829797	-394.82011
A_3T_3	-921.1889	-467.181355	-453.993295
$^{OXO}G_4C_2$	-1012.47815	-617.629398	-394.819362
A_5T_1	-921.192215	-467.181968	-453.993245
A_1T_5	-921.190481	-467.1820619	-453.9938144
$(S)Sp^{SYN}{}_2C_4$	-1087.659783	-692.8223505	-394.8199876
A_3T_3	-921.191779	-467.1816852	-453.9927506
$^{OXO}G_4C_2$	-1012.478496	-617.629962	-394.8197273
A_5T_1	-921.191974	-467.1818355	-453.9931953
A_1T_5	-921.180699	-467.181314	-453.993971
$(R)Sp^{ANTI}{}_2C_4$	-1087.655006	-692.829797	-394.82011
A_3T_3	-921.191897	-467.181355	-453.993295
$^{OXO}G_4C_2$	-1012.477165	-617.629398	-394.819362
A_5T_1	-921.192216	-467.181968	-453.993245
A_1T_5	-921.191237	-467.1809357	-453.9933699
$(R)Sp^{SYN}{}_2C_4$	-1087.652925	-692.8282118	-394.8195375
A_3T_3	-921.187019	-467.1786213	-453.9936995
$^{OXO}G_4C_2$	-1012.477361	-617.6291809	-394.8195375
A_5T_1	-921.192292	-467.1820153	-453.9932948

Table S3. The energies (in Hartree) of Neural, Vertical Cation, Adiabatic Cation, and Vertical Neutral forms of base pairs extracted from *ds*-oligonucleotides calculated at the M06-2x/6-31++G** level of theory in the aqueous phase.

oligo-(S)Sp^{SYN}	**Neutral**	**Vertical Cation**	**Adiabatic Cation**	**Vert Neutral**
**A_1T_5**	-921.18123	-920.940447	-920.941744	-921.180647
**$(S)S_P{}^{SYN}{}_2C_4$**	-1087.658814	-1087.404804	-1087.405122	-1087.654646
**A_3T_3**	-921.1889	-920.93883	-920.950062	-921.189244
**$^{OXO}G_4C_2$**	-1012.47815	-1012.260824	-1012.273824	-1012.460078
**A_5T_1**	-921.192215	-920.945091	-920.946754	-921.191352
oligo-(S)Sp^{SYN}		**Vertical Anion**	**Adiabatic Anion**	**Vert Neutral**
**A_1T_5**		-921.23899	-921.240177	-921.182117
**$(S)S_P{}^{SYN}{}_2C_4$**		-1087.706041	-1087.707997	-1087.658794
**A_3T_3**		-921.240814	-921.240944	-921.188559
**$^{OXO}G_4C_2$**		-1012.534422	-1012.550246	-1012.458968
**A_5T_1**		-921.244435	-921.242996	-921.191195
oligo-(S)Sp^{ANTI}	**Neutral**	**Vertical Cation**	**Adiabatic Cation**	**Vert Neutral**
**A_1T_5**	-921.18123	-920.940447	-920.941744	-921.180647
**$(S)S_P{}^{ANTI}{}_2C_4$**	-1087.658814	-1087.404804	-1087.405122	-1087.654646
**A_3T_3**	-921.1889	-920.93883	-920.950062	-921.189244
**$^{OXO}G_4C_2$**	-1012.47815	-1012.260824	-1012.273824	-1012.460078
**A_5T_1**	-921.192215	-920.945091	-920.946754	-921.191352
oligo-(S)Sp^{ANTI}		**Vertical Anion**	**Adiabatic Anion**	**Vert Neutral**
**A_1T_5**		-921.23899	-921.240177	-921.182117
**$(S)S_P{}^{ANTI}{}_2C_4$**		-1087.706041	-1087.707997	-1087.658794
**A_3T_3**		-921.240814	-921.240944	-921.188559
**$^{OXO}G_4C_2$**		-1012.534422	-1012.550246	-1012.458968
**A_5T_1**		-921.244435	-921.242996	-921.191195
oligo-(R)Sp^{ANTI}	**Neutral**	**Vertical Cation**	**Adiabatic Cation**	**Vert Neutral**
**A_1T_5**	-921.180699	-920.932421	-920.932835	-921.180882
**$(R)S_P{}^{ANTI}{}_2C_4$**	-1087.655006	-1087.373482	-1087.374405	-1087.655891
**A_3T_3**	-921.191897	-920.948787	-920.948816	-921.191947
**$^{OXO}G_4C_2$**	-1012.477165	-1012.25913	-1012.274094	-1012.465882
**A_5T_1**	-921.192216	-920.944936	-920.94672	-921.192224
oligo-(R)Sp^{ANTI}		**Vertical Anion**	**Adiabatic Anion**	**Vert Neutral**
**A_1T_5**		-921.236175	-921.23601	-921.180647
**$(R)S_P{}^{ANTI}{}_2C_4$**		-1087.703499	-1087.703911	-1087.654646
**A_3T_3**		-921.244904	-921.241016	-921.189244
**$^{OXO}G_4C_2$**		-1012.533121	-1012.549461	-1012.460078
**A_5T_1**		-921.244754	-921.244209	-921.191352
oligo-(R)Sp^{SYN}	**Neutral**	**Vertical Cation**	**Adiabatic Cation**	**Vert Neutral**
**A_1T_5**	-921.191237	-920.943241	-920.942845	-921.19114
**$(R)S_P{}^{ANTI}{}_2C_4$**	-1087.652925	-1087.398151	-1087.39884	-1087.65396
**A_3T_3**	-921.187019	-920.935522	-920.945936	-921.190199
**$^{OXO}G_4C_2$**	-1012.477361	-1012.260165	-1012.273866	-1012.465629
**A_5T_1**	-921.192292	-920.945663	-920.947209	-921.192329
oligo-(R)Sp^{SYN}		**Vertical Anion**	**Adiabatic Anion**	**Vert Neutral**
**A_1T_5**		-921.242637	-921.242219	-921.190562
**$(R)S_P{}^{ANTI}{}_2C_4$**		-1087.701744	-1087.701492	-1087.651479
**A_3T_3**		-921.239522	-921.239034	-921.186762
**$^{OXO}G_4C_2$**		-1012.533543	-1012.533577	-1012.474971
**A_5T_1**		-921.244988	-921.259697	-921.175087

Table S4. Hirshfeld charge and spin distribution in the shape of ds-oligo presented in Table 1, only nucleosides bases were taken into consideration, calculated at the M06-2x/6-31++G** level of theory in the aqueous phase. Vertical Cation (VC^{NC}) (NE-non-equilibrated), Vertical Cation (VC^{EQ}) (EQ-equilibrated), Vertical Anion (VA^{NE}), Vertical Anion (VA^{EQ}), Adiabatic Cation (AC), Adiabatic Anion (AA) and Vertical Neutral from Cation (VNC^{NE}), Vertical Neutral from Cation (VNC^{EQ}), Vertical Neutral from Anion (VNA^{NE}), Vertical Neutral from Anion (VNA^{EQ})

oligo-(R)Sp^{ANTI}							
	**Neutral**	**VC^{NE}**		**VC^{EQ}**		**AC**	
	**Charge**	**Charge**	**Spin**	**Charge**	**Spin**	**Charge**	**Spin**
**A_1T_5**	-0.12	-0.10	0.00	-0.12	0.00	-0.10	0.00
**$(R)Sp^{ANTI}{}_2C_4$**	0.07	0.09	0.00	0.09	0.00	0.09	0.00
**A_3T_3**	0.05	0.18	0.10	0.16	0.08	0.12	0.06
**$^{OXO}G_4C_2$**	-0.01	0.76	0.87	0.80	0.90	0.83	0.92
**A_5T_1**	0.00	0.07	0.02	0.06	0.02	0.06	0.02
oligo-(R)Sp^{ANTI}							
	**VA^{NE}**		**VA^{EQ}**		**AA**		
	Charge	Spin	Charge	Spin	Charge	Spin	
**A_1T_5**	-0.15	0.01	-0.12	0.00	-0.12	0.00	
**$(R)Sp^{ANTI}{}_2C_4$**	-0.11	0.18	0.06	0.00	0.05	0.00	
**A_3T_3**	-0.48	0.58	-0.05	0.09	-0.04	0.03	
**$^{OXO}G_4C_2$**	-0.17	0.16	-0.56	0.60	-0.83	0.93	
**A_5T_1**	-0.09	0.07	-0.32	0.31	-0.06	0.03	
oligo-(R)Sp^{SYN}							
	**Neutral**	**VC^{NE}**		**VC^{EQ}**		**AC**	
	**Charge**	**Charge**	**Spin**	**Charge**	**Spin**	**Charge**	**Spin**
**A_1T_5**	0.03	0.00	0.03	0.00	0.02	0.00	0.03
**$(R)Sp^{SYN}{}_2C_4$**	-0.05	0.00	-0.06	0.00	-0.07	0.00	-0.03
**A_3T_3**	0.00	0.07	0.12	0.06	0.12	0.05	0.08
**$^{OXO}G_4C_2$**	0.02	0.90	0.82	0.91	0.85	0.93	0.85
**A_5T_1**	0.00	0.02	0.08	0.02	0.07	0.02	0.07
oligo-(R)Sp^{SYN}							
	**VA^{NE}**		**VA^{EQ}**		**AA**		
	Charge	Spin	Charge	Spin	Charge	Spin	
**A_1T_5**	0.00	0.01	0.00	0.02	0.00	0.03	
**$(R)Sp^{SYN}{}_2C_4$**	0.01	-0.12	0.00	-0.10	0.00	-0.07	
**A_3T_3**	0.32	-0.27	0.08	-0.06	0.00	-0.02	
**$^{OXO}G_4C_2$**	0.60	-0.51	0.68	-0.60	0.02	-0.10	
**A_5T_1**	0.07	-0.10	0.24	-0.25	0.98	-0.84	

**Table S4 continuous**						
oligo-(S)Sp^{ANTI}						
	**Neutral**	**VC^{NE}**		**VC^{EQ}**		**AC**
	**Charge**	**Charge**	**Spin**	**Charge**	**Spin**	**Charge**	**Spin**
**A_1T_5**	0.19	0.20	0.00	0.19	0.00	0.21	0.00
**(*S*)$S_P{}^{ANTI}{}_2C_4$**	-0.22	-0.20	0.00	-0.21	0.00	-0.13	0.00
**A_3T_3**	-0.04	0.06	0.07	0.05	0.06	0.06	0.07
**$^{OXO}G_4C_2$**	0.07	0.85	0.90	0.89	0.91	0.80	0.90
**A_5T_1**	0.01	0.08	0.03	0.08	0.03	0.06	0.02
oligo-(S)Sp^{ANTI}						
	**VA^{NE}**		**VA^{EQ}**		**AA**		
	Charge	Spin	Charge	Spin	Charge	Spin	
**A_1T_5**	-0.57	0.79	0.18	0.00	0.11	0.00	
**(*S*)$S_P{}^{ANTI}{}_2C_4$**	-0.42	0.19	-0.23	0.00	-0.21	0.00	
**A_3T_3**	-0.06	0.01	-0.13	0.07	-0.06	0.03	
**$^{OXO}G_4C_2$**	0.06	0.00	-0.65	0.78	-0.77	0.94	
**A_5T_1**	0.00	0.00	-0.17	0.15	-0.06	0.03	
oligo-(S)Sp^{SYN}						
	**Neutral**	**VC^{NE}**		**VC^{EQ}**		**AC**	
	**Charge**	**Charge**	**Spin**	**Charge**	**Spin**	**Charge**	**Spin**
**A_1T_5**	-0.04	0.00	-0.04	0.00	-0.05	0.00	-0.03
**(*S*)$S_P{}^{SYN}{}_2C_4$**	-0.20	0.00	-0.01	0.00	-0.02	0.00	-0.02
**A_3T_3**	0.00	0.05	0.12	0.04	0.11	0.04	0.13
**$^{OXO}G_4C_2$**	0.10	0.93	0.86	0.94	0.89	0.94	0.86
**A_5T_1**	0.14	0.02	0.07	0.02	0.07	0.02	0.06
oligo-(S)Sp^{SYN}						
	**VA^{NE}**		**VA^{EQ}**		**AA**		
	Charge	Spin	Charge	Spin	Charge	Spin	
**A_1T_5**	0.35	-0.42	0.01	-0.07	0.91	-0.85	
**(*S*)$S_P{}^{SYN}{}_2C_4$**	0.56	-0.54	0.03	-0.08	0.09	-0.19	
**A_3T_3**	0.09	-0.06	0.22	-0.19	0.00	0.06	
**$^{OXO}G_4C_2$**	0.01	0.02	0.66	-0.57	0.00	-0.01	
**A_5T_1**	0.00	-0.01	0.08	-0.10	0.00	-0.01	

Table S5. The Energies: Ground (E^{GR}) and Excitation (E^{EX}) state energies and Excitation and HOMO Energies as well as corresponding Dipole Moments Ground, Excitation, and Transition (DM^{G}, DM^{EX}, D_{12}) in Debays of neighbor base pair extracted from selected dimmers of *ds*-oligonucleotides, calculated at the M06-2x/6-31++G** level of theory in the aqueous phase using the DFT or TD-DFT methodology.

**$d[A_1(S)S_P^{SYN}{}_2A_3{}^{O}G_4A_5]*[T_1C_2T_3C_4T_5]$**										
**SYSTEM**	**Base Pair Dimer**	**E^{GR}**	**DM^{GR}**	**E^{EX}**	**DM^{EX}**	**D_{12}**	**E^{HOMO}**	**E^{HOMO-1}**	**E^{LUMO}**	**E^{LUMO+1}**
**(*S*)S_P^{SYN}**	**A_1 || (*S*)$S_P^{SYN}{}_2$**	-2008,866677	12,89	-2008,866677	12,96	15,41	-0,2761	-0,2965	-0,0191	-0,0111
	**(*S*)$S_P^{SYN}{}_2$ || A_3**	-2008,878962	15,44	-2008,878962	15,74	6,29	-0,2867	-0,2963	-0,0193	-0,0141
	**A_3 || $^{oxo}G_4$**	-1933,695473	16,23	-1933,695473	14,43	6,22	-0,2536	-0,2821	-0,0106	-0,0179
	**$^{oxo}G_4$ || A_5**	-1933,695676	16,36	-1933,695676	14,68	4,70	-0,2535	-0,2833	-0,0176	-0,0136
**$d[A_1(S)S_P^{ANTI}{}_2A_3{}^{O}G_4A_5]*[T_1C_2T_3C_4T_5]$**										
**(*S*)S_P^{ANTI}**	**A_1 || (*S*)$S_P^{ANTI}{}_2$**	-2008,864459	17,26	-2008,731714	17,69	14,09	-0,2912	-0,2976	-0,0214	-0,0177
	**(*S*)$S_P^{ANTI}{}_2$ || A_3**	-2008,869509	16,07	-2008,736415	16,51	7,65	-0,2874	-0,2934	-0,0155	-0,0125
	**A_3 || $^{oxo}G_4$**	-1933,693424	15,77	-1933,568558	14,05	3,52	-0,2550	-0,2895	-0,0187	-0,0145
	**$^{oxo}G_4$ || A_5**	-1933,696264	16,19	-1933,570829	14,44	5,49	-0,2532	-0,2836	-0,0180	-0,0146
**$d[A_1(R)S_P^{SYN}{}_2A_3{}^{O}G_4A_5]*[T_1C_2T_3C_4T_5]$**										
**(*R*)S_P^{SYN}**	**A_1 || (*R*)$S_P^{ANTI}{}_2$**	-1933,566092	18,35	-1933,566092	14,36	0,60	-0,2534	-0,2880	-0,0170	-0,0151
	**(*R*)$S_P^{ANTI}{}_2$ || A_3**	-1933,580963	18,26	-1933,580963	15,23	0,91	-0,2884	-0,2936	-0,0154	-0,0130
	**A_3 || $^{oxo}G_4$**	-1933,692665	15,94	-1933,692665	14,16	2,95	-0,2538	-0,2908	-0,0186	-0,0162
	**$^{oxo}G_4$ || A_5**	-1933,694296	16,42	-1933,694296	14,81	3,95	-0,2532	-0,2834	-0,0180	-0,0146
**$d[A_1(R)S_P^{ANTI}{}_2A_3{}^{O}G_4A_5]*[T_1C_2T_3C_4T_5]$**										
**(*R*)S_P^{ANTI}**	**A_1 || (*R*)$S_P^{ANTI}{}_2$**	-2008,864021	10,46	-2008,731044	10,61	2,70	-0,2815	-0,2918	-0,0156	-0,0137
	**(*R*)$S_P^{ANTI}{}_2$ || A_3**	-2008,864346	9,29	-2008,731658	9,37	6,32	-0,2797	-0,2959	-0,0186	-0,0152
	**A_3 || $^{oxo}G_4$**	-1933,696486	16,97	-1933,571567	15,26	4,22	-0,2516	-0,2834	-0,0171	-0,0155
	**$^{oxo}G_4$ || A_5**	-1933,693638	16,24	-1933,566604	14,61	4,27	-0,2542	-0,2840	-0,0181	-0,0140

Table S6. The Energies: Ground (E^{GR}) and Excitation (E^{EX}) state energies and Excitation and HOMO Energies as well as corresponding Dipole Moments Ground, Excitation, and Transition (DM^{G}, DM^{EX}, D_{12}) in Debays of distal base pair extracted from selected trimmers of *ds*-oligonucleotides, calculated at the M06-2x/6-31++G** level of theory in the aqueous phase using the DFT or TD-DFT methodology

**d[A_1(*S*)$S_P^{SYN}$$_2$$A_3^O$$G_4$$A_5$]*[$T_1C_2T_3C_4T_5$]**										
**SYSTEM**	**Base Pair Dimer**	**E^{GR}**	**DM^{GR}**	**E^{EX}**	**DM^{EX}**	**D_{12}**	**E^{HOMO}**	**E^{HOMO-1}**	**E^{LUMO}**	**E^{LUMO+1}**
**(*S*)S_P^{SYN}**	**A_1||A_3**	-1842.383032	4.51	-1842.249997	4.92	11.89	-0.282895	-0.283368	-0.018317	-0.013395
	**(*S*)S_P^{SYN} ||G_4**	-2100.139157	25.2678	-2100.011373	23.73	6.29	-0.256563	-0.301537	-0.020684	-0.015952
	**A_3||A_5**	-1842.384519	3.94	-1842.250837	3.96	9.89	-0.283446	-0.286532	-0.018577	-0.012747
**d[A_1(*S*)$S_P^{ANTI}$$_2$$A_3^O$$G_4$$A_5$]*[$T_1C_2T_3C_4T_5$]**										
**(*S*)S_P^{ANTI}**	**A_1||A_3**	-1842.370956	8.18	-1842.237925	8.48	12.13	-0.280328	-0.289601	-0.020041	-0.015865
	**(*S*)S_P^{ANTI} ||G_4**	-2100.137574	29.73	-2100.00988	27.95	6.08	-0.256498	-0.296787	-0.021359	-0.015873
	**A_3||A_5**	-1842.381995	3.79	-1842.24847	3.72	9.22	-0.286656	-0.289621	-0.019463	-0.013247
**d[A_1(*R*)$S_P^{SYN}$$_2$$A_3^O$$G_4$$A_5$]*[$T_1C_2T_3C_4T_5$]**										
**(*R*)S_P^{SYN}**	**A_1||A_3**	-1842.379081	4.46	-1842.245237	4.60	10.39	-0.287347	-0.291009	-0.018293	-0.013905
	**(*R*)S_P^{SYN} ||G_4**	-2100.131238	33.1	-2100.00369	31.53	6.58	-0.256407	-0.297805	-0.021016	-0.016374
	**A_3||A_5**	-1842.380153	3.38	-1842.246654	3.41	10.21	-0.286187	-0.291176	-0.019423	-0.0134
**d[A_1(*R*)$S_P^{ANTI}$$_2$$A_3^O$$G_4$$A_5$]*[$T_1C_2T_3C_4T_5$]**										
**(*R*)S_P^{ANTI}**	**A_1||A_3**	-1842.384285	3.76	-1842.384285	3.97	4.13	-0.281472	-0.28527	-0.018637	-0.012621
	**(*R*)S_P^{ANTI} ||G_4**	-1949.731085	17.67	-1949.731085	15.62	2.45	-0.257209	-0.264934	-0.019173	-0.01485
	**A_3||A_5**	-1842.3844	4.84	-1842.3844	4.86	4.09	-0.281545	-0.286809	-0.018885	-0.013336
